# Another Case of Priapism from Nasal Reflex

**Published:** 1906-07

**Authors:** Arthur G. Hobbs

**Affiliations:** Atlanta, Ga.


ATLANTA

### Journal-Record of Medicine

Successor to Atlanta Medical and Surgical Journal, Established 1855, and Southern Medical Record, Established 1870.

OWNED BY THE ATLANTA MEDICAL JOURNAL CO.

Published Monthly.

VOL. VIII. JULY, 1906. No. 4.

BERNARD WOLFF, M.D., EDITOR, Nos. 319-20 Prudential.

M. B. HUTCHINS, M.D., BUSINESS MANAGER, 1007-1008 Century Bldg.

E. G. BALLENGER, M.D., ASSOCIATE EDITOR AND ASSISTANT MANAGER.

### ORIGINAL COMMUNICATIONS.

### ANOTHER CASE OF PRIAPISM FROM NASAL REFLEX.*

BY ARTHUR G. HOBBS, M.D.,

ATLANTA, GA.

INDEX THROUGH

This, my third case, is the seventh one on record. The two others that I saw in 1888 were the first recorded. Four more cases only have been reported, like mine in all essential respects. As these three cases came under my observation purely by accident, I make no claim to any merit for myself other than to have been the first to record them as unique complications—as phenomenal cases of nervo-pathological reflexes.

An electrician, age thirty-three, unmarried, came to my office November 10, 1904, in much discomfort and pain from a nasal stenosis and its consequent frontal headache. He had been confined to his room for a few days from a strabismus operation, but I had dismissed him after having removed the stitches on Novem-

* Read before the Atlanta Society of Medicine, April 5, 1906.

ber 6. He awoke on the morning of the 7th with a nasal stuffiness and a dull, heavy pain across the brow. The pain grew worse during the day, as the nasal pressure increased; after getting to sleep that night he was awakened early on the morning of the 8th with much pain in the head and face, and also, to his surprise, *with a priapism.* "No other pain had ever equaled it," he said. The priapism increased in severity, except for the temporary remissions when he was completely under the influence of an opiate, until he came to my office on the morning of November 10th with the hope only of having his nasal and head pains relieved. His face looked drawn and pinched from the suffering he had gone through for more than three days with a literally closed nose and its consequent neuralgic pains. But added to this (enough in itself) was his two days of an acute priapism. This man had had all the naturally suggestive treatments for his condition, such as hot and cold applications, opiates, chloroform, the suprarenal extracts internally and even apomorphia to the extent of emesis.

He had gone through all of this at the hands of his relative, Dr. A. R. S., of Louisville, Ky., who, by chance, was rooming with him during a few days stop-over in Atlanta while on his way to Florida.

The doctor came with the patient from his rooms, only a few blocks from my office, and told me that no treatment had given him more than a partial and temporary relief.

Examination showed a completely occluded nasal passage, with a pressure so great as to cause an outward bulging of the turgescent nasal tissues.

The treatment. I first gave him 20 drops of adnephrin, and then pellets of cotton, saturated in a strong solution of adnephrin and cocaine, were insinuated, then larger and still larger pellets at intervals of a few minutes, until the saturated probe could gain an entrance, and finally a spray of a weaker solution. After about thirty minutes the relief of having the nasal lumen restored was marked by a decided change in the facial expression, but the patient's joy was yet more apparent when he exclaimed that the swelling below was passing away and that the pain there was gone.

Some enlargement of the lower organ remained, but there was no longer any tension in the corpora cavernosa. After a six hours' sleep, the patient awoke with some stuffiness of the nose and a slight return of the pain below, but when I repeated the treatment, late the same day, the relief was still more decided. On the next day there was no return of the priapism, and even the soft enlargement was gone. Some erectile tissue swelling over the turbinates remained, that felt like tense rubber to the cotton probe for some days; but daily applications of a weak organic silver solution with adnephrin gave a comparatively normal nasal lumen, although three months later, in February, 1905, I had to make some galvano-cautery punctures to reduce the resulting hypertrophies in the lower turbinates.

During the interim to this date of almost a year and a half there has been no return of the priapism, nor has there been any recurrence of the nasal symptoms for more than a year.

In short, the first symptoms in this case, on November 6th, suggested only an acute rhinitis. November 7th there was increased nasal stenosis, with reflex head pains and some pyrexia. November 8th, symptoms all aggravated, especially the frontal pains, but no fever. Then came the priapism, rare, unexpected, and naturally considered a coincidence, as it probably does not complicate even so great a turbinal turgescence as this patient had once in many thousand times. The immediate relief to the priapism after the reduction of the nasal stenosis leaves no doubt of its complication in this case, and the same is true of the two other cases that I have recorded.

Then why this seeming phenomenon? Shall we not leave the answer to the pathologist—to the neuro-pathologist—who deals in curios?

In 1889 I casually mentioned my two first cases before the Atlanta Academy of Medicine, in a discussion on an allied subject, and soon afterwards described them in a short published paper.

These two cases were the basis of a paper I read again, with some corrections to the subject, eight years later before the American Laryngological, Rhinological and Otological Association in New Orleans.

The First Case: A young bank cashier (L. R.), was driven to my office in a closed cab wearing an overcoat on an August day in 1888 to conceal his priapism. He had run the gauntlet of all the then known treatments for his priapism, which had been unremitting for two days. He looked haggard and pale, and so weak that his room-mate had to come with him. The object of his visit to me was to be relieved of an acute rhinitis and an almost complete nasal stenosis.

I make only a short synopsis.

After a half hour's persistence with cotton pellets saturated in a strong solution of cocaine, repeated at intervals, then with a weaker spray, until he showed its systemic effects, his nose began to open and the rigidity of the priapism to decrease. Yet there were slight recurrences of the pain in the lower organ for two or three days before a complete relaxation was reached.

It was rather singular that I should have seen the second case during the same year, when it was sixteen years later when I saw the third one. I will not quote the description of the case, as it was so nearly like the first in the symptoms, treatments and results. At that time we had no suprarenal extracts.

The corollaries of this subject are most interesting. Many of them have been suggested to me through the forty-odd letters that I received from doctors in this country and from abroad after the publications of these first two cases.

An apparently unaccountable sneeze may come to some persons merely through an erotic thought, when accompanied by the desire and the anticipation of its possible fruition. All whom I have questioned on this point say that the sneezing reflex passes off long before the more important erectile organ has reached its limit of distention. I do not doubt, from the accidental way in which some such instances have come to my knowledge, that many more men than we know of, and possibly some women, are endowed with this propensity.

From the descriptions, the sneeze is characteristic and quite unlike the first announcement of a cold. One gentleman has described his own case to me more graphically than I can repeat it. While walking with a lady, he said, one evening, his wife, who was but a short distance in the rear, was surprised, as she soon afterwards took occasion to tell him, to hear a succession of sneezes with which she thought she was quite familiar. Later, however, she was amused at her own mistake, and no doubt delighted, too, when he made her realize that what she had heard was only the ordinary, commonplace, bad cold sneeze (?) that any man might unfortunately contract at the wrong time! A younger man has told me that he was often mortified and placed at his wit's end in making excuses to his sweetheart on account of his susceptibility, when with her, to drafts (?) that she could in no way discover.

Several men have acknowledged that this little telltale nasal reflex had been their principal cause for consulting me. A physician, a member of this Society, once very graphically described to me his own experience with this reflex. The sneeze came to him, he said, so insiduously that he could scarcely realize that he had had it. Indeed, his wife was familiar with the characteristics of this "forerunner" long before he could himself acknowledge it. It would seem that none of the means that are ordinarily resorted to can suppress this expletive. It simply means that "Barkis is willin'," or that he would like to be; at least, that such an idea exists in the brain of the subject, whether or not it can be well and effectually reflected in the proper direction. In all cases, without an exception, it would appear that the effect on the sneezing area has come and gone before the mental reflex has had time to reach its proper state of erectile perfection. A sneeze is one of the typical examples of nerve reflex, and while the neurologist can only construct a beautiful theory to explain its incentive and its conductive factors, the pathologist tells us that the final result is brought about through the swelling of the erectile tissue covering the turbinal bones. The histological similarity between the turbinal tissues and the cavera cavernosa is quite sufficient to cause no wonder or surprise that a mental impression that excites the one should also excite the other, even within physiological bounds, although we are accustomed to regard the one as an entirely physiological function and the other as distinctively pathological, since it passes too far beyond all the physiological necessities.

The instances just cited prove, however, that it is only the turbinal erectile tissue that possesses the dual capacity of reflecting either a physiological or a pathological stimulus. Also that its response to a mental stimulus always precedes the more important effect below, and is gone before the distention of the cavera cavernosa is complete. May it be that a priapism always begins as a physiological condition and ends as a pathological one?

Nature's seeming vagaries are naturally amusing when rare, but when frequent the sense of the ludicrous is dulled. We have learned to accept without question—as a matter of course—the many physiological involition reflexes as we do those of volition. But the most advanced pathologists are still groping in the dark when they approach pathological nerve reflexes. For this reason the least suggestion in that direction is worthy of record.

I trust that all will understand that it has been far from my intention to deal with this subject flippantly, or even lightly, since it is fraught with so much scientific suggestiveness.

*805-807-809 English-American Building.*

DISCUSSION OF DR. HOBBS' PAPER.

*Dr. Hutchins:* I do not know if Dr. Hobbs has ever noticed or had called to his attention the existence of the nasal reflex phenomena appearing in the lower animals. Possibly he is familiar with the peculiar sneeze of the goat at certain times.

*Dr. Cartledge:* There was a case of priapism reported at this Society by Dr. White some time ago. I would like to know what the outcome was.

*Dr. Hobbs:* The goat's sneeze, or snort, has just been mentioned as an announcement by his goatship that he was ready for the fray. If the doctor had read the forty-five or fifty letters that came to me after the publication of my first two cases of reflex priapism, he would have seen that such instances of physiological nasal reflex in the lower animals are frequent. I could have talked for an hour on these physiological corollaries, based upon these letters, perhaps to the amusement of all. But our real question is that of pathological nerve reflexes, where our pathologists are groping in the dark. This has been my only excuse for alluding to the few physiological corollaries to this subject in the body of my report.

I was asked to go to see a case of priapism by Drs. White and Champion a few months ago. The case has since been reported before this society. But it was not a case of nasal reflex priapism, as there was no indication of the involvement of the erectile turbinal tissues. I could only suggest suprarenal extracts internally and apomorphia to the extent of emesis.

(P. S.—I would be glad to receive any letters on this subject from any one who has an analogous case. Due credit will be given in the future publications that I may make on this subject. A. G. H.)

